# Exercising Tactically for Taming Postmeal Glucose Surges

**DOI:** 10.1155/2016/4045717

**Published:** 2016-03-17

**Authors:** Elsamma Chacko

**Affiliations:** Connecticut Valley Hospital, 100 Silver Street, Middletown, CT 06457, USA

## Abstract

This review seeks to synthesize data on the timing, intensity, and duration of exercise found scattered over some 39 studies spanning 3+ decades into optimal exercise conditions for controlling postmeal glucose surges. The results show that a light aerobic exercise for 60 min or moderate activity for 20–30 min starting 30 min after meal can efficiently blunt the glucose surge, with minimal risk of hypoglycemia. Exercising at other times could lead to glucose elevation caused by counterregulation. Adding a short bout of resistance exercise of moderate intensity (60%–80%  VO_2max_) to the aerobic activity, 2 or 3 times a week as recommended by the current guidelines, may also help with the lowering of glucose surges. On the other hand, high-intensity exercise (>80%  VO_2max_) causes wide glucose fluctuations and its feasibility and efficacy for glucose regulation remain to be ascertained. Promoting the kind of physical activity that best counters postmeal hyperglycemia is crucial because hundreds of millions of diabetes patients living in developing countries and in the pockets of poverty in the West must do without medicines, supplies, and special diets. Physical activity is the one tool they may readily utilize to tame postmeal glucose surges. Exercising in this manner does not violate any of the current guidelines, which encourage exercise any time.

## 1. Introduction

The value of exercise in keeping body and mind in good health was an article of faith among physicians of the time of Hippocrates. More recently, before the insulin era began, doctors had been prescribing exercise for diabetes. Now, decades of clinical studies have established that blood glucose levels are sensitive to exercise timing, intensity, duration, and frequency [1, 2]. A 2013 review [1] concluded that postmeal exercise was better than premeal exercise for managing hyperglycemia.

In fact, it is no longer adequate to categorize exercise timing into just premeal and postmeal in diabetes studies. The postmeal period is an eventful stretch of time [3], unlike the usually quiet premeal period. In the healthy human body, insulin and the counterregulatory hormones work in tandem to keep blood glucose levels within normal bounds [4]. Glucose levels peak around the 1-hour mark after meal and decline to the premeal level in two to four hours [3]. Glucose levels in healthy people may not go above 140 mg/dL. In people with diabetes, a substantial peak could develop with the height, width, slopes, and peaking time showing wide variations based on several factors, including the state of diabetes, meal size, meal composition, level of activity, and medications.

This paper combs published data looking for ways to tame the postmeal glucose surge [5] through exercise without triggering hypoglycemia [1, 6, 7]. The assembled database consists of 39 articles [8–46] that report timing, intensity, and duration of the exercise activity. The feeding cycle is perceived as consisting of four time segments: before meal, early postprandial (0–29 min after meal,) midpostprandial (30 min to 120 min after meal), and late postprandial (>120 min after meal). Exercise intensity in particular is measured using a large number of variables: %VO_2max_ (maximal oxygen uptake), %VO_2peak_ (peak oxygen uptake), %HR_max_ (heart rate max, calculated as 220 − age), %HRR (heart rate reserve), and %W_max_ (maximal power output) are some of the commonly used units. The categorization of exercise intensity, a continuous variable, into light (<60%  VO_2max_), moderate (60%–80%  VO_2max_), and high (>80%  VO_2max_), is dictated by the distribution of the values found in the assembled database. These categories serve the purpose extant here and may conform only approximately to others used elsewhere.

## 2. Search and Selection

The articles at the heart of this review came from a related project concluded in 2014 [2]. Iterative searches of MEDLINE using the search terms “exercise timing,” “post meal exercise,” “pre meal exercise,” “postprandial exercise,” “preprandial exercise,” “post-absorptive exercise,” and “HIT exercise.” Modifiers used in the database search were “high-intensity,” “intense,” “moderate,” “light,” “glucose,” “metabolic syndrome,” “obesity,” and “diabetes.” Other relevant studies came from the reference lists of landmark articles and from a hand search of appropriate journals and reviews.

Studies of blood glucose response to a single bout of light to moderate exercise (<80%  VO_2max_) during premeal or postmeal period found their way into the database if intensity and duration were also among the study variables. The health benefits of high-intensity interval training (HIT) were well established through recent reviews [47–53]. A few articles from this group that showed glucose surges were included. Excluded were studies focused on glucose response to different meals or on medications along with exercise. This search did not use any cut-off dates.

Out of the 39 articles [8–46] found in Table 1, 23 studied the glucose response to light to moderate (<80%  VO_2max_) intensity exercise, before meal [8–21] or after meal [22–30]. Eight studied high-intensity (>80%  VO_2max_) exercise, before meal or after meal [31–38]. One study used premeal and postmeal resistance exercise [39]. The remaining seven articles compared effects such as training in fasted versus fed state [40–43], different intensities [44, 45], and different durations [46].

## 3. Results

### 3.1. Premeal Period

In general, glucose levels were steady during light to moderate premeal exercise and there was little risk of hypoglycemia in a wide range of populations: men with type 2 diabetes, individuals with insulin resistance, and healthy men and healthy women [8–10, 17, 19]. When glucose did go down, the extent of the decline depended on its preexercise level [8]. Light to moderate premeal exercise, however, led to postprandial glucose elevations immediately following the exertion. This effect was consistently seen in men with metabolic syndrome, men and women with type 2 diabetes, men and women with prediabetes, individuals with type 1 diabetes, and people with insulin resistance [11–15]. Melton and colleagues observed that moderate premeal exercise in people with prediabetes did not decrease oxidative stress in people with prediabetes; oxidative stress is closely linked to glucose surges [16]. Borer and colleagues found improved fasting glucose levels in healthy postmenopausal women after they completed two two-hour bouts of light premeal exercise [18]. The authors inferred liver glycogen depletion from markedly elevated levels of 3-hydroxybutyrate, free fatty acids, cortisol, and glucagon during the premeal exercise. A direct measurement showed that hepatic glucose production remained elevated during moderate premeal exercise [19]. At relatively high intensities (75%  VO_2max_) premeal exercise also showed a delayed, modest insulin sensitivity improvement according to glucose area-under-the-curve (g-AUC) calculations [20, 21].

High-intensity premeal exercise resulted in significant postexertion glucose elevation in healthy men, men with type 2 diabetes, and people with type 1 diabetes [31–34]. These studies reported elevated levels of epinephrine, norepinephrine, glucagon, lactate, and pyruvate and depressed levels of glycerol, free fatty acids, and 3-hydroxybutyrate during the exercise activity [31, 33, 34, 54]. Some improvement in insulin sensitivity that lasted for up to 24 hours was also observed [15, 31, 47–51]. In people with type 1 diabetes nocturnal hypoglycemia was seen after high-intensity premeal exercise [6, 47, 51].

### 3.2. Early Postprandial Period

When light activity began 15 minutes after the start of meal, glucose levels kept rising during the first 15 minutes of the exercise at the end of which, starting at 30 min after meal, glucose started to go down [22–24]. The secondary peak that formed after the activity stopped was prominent, signaling suboptimal blunting of the glucose peak. The subjects in these studies were healthy men and women and people with type 1 diabetes. One study using moderate intensity (65%  VO_2max_) exercise at 15 min after meal reported that a one-third reduction in postmeal glucose elevation occurred when g-AUC was compared between exercising and nonexercising subjects [25].

### 3.3. Midpostprandial Period

Twenty-two midpostprandial studies using light to moderate exercise demonstrated glucose lowering with the activity [8–14, 17–19, 26–30, 44–46, 55–59]. These efforts spanned virtually all demographics of interest in diabetes studies: healthy women, healthy men, men with type 2 diabetes, women with type 2 diabetes, people with type 2 diabetes on insulin, men with metabolic syndrome, patients with type 1 diabetes, and healthy postmenopausal women. When the light to moderate exercise started 30 [12, 19, 26–28] or 45 min [17, 29, 44] after meal, there was good blunting of the postmeal glucose peak: for example, Larsen and colleagues estimated that the postprandial glucose surge was diminished by 50%, according to g-AUC calculations, when the subjects exercised for 45 min starting at 45 min after meal, at 53%  VO_2max_ [29].

High-intensity postmeal exercise during midpostprandial period did not result in significant postexertion glucose elevation in contrast to the glucose elevation found in the case of high-intensity premeal exercise. A delayed insulin sensitivity improvement was discernible [35–38]. The subjects in these studies were adults with type 2 diabetes, healthy adults, and inactive obese men.

### 3.4. Late Postprandial Period

The glycemic effect of light exercise during the late postprandial period (4.5 h) showed postprandial glucose elevation in people with prediabetes, reminiscent of the premeal effect [13]. Sasaki and colleagues demonstrated that the glycemic response to premeal exercise was nearly identical to that of the 4 h postmeal exercise in young trained athletes [56].

High-intensity exercise at three hours after the meal in healthy men showed a significant postexertion glucose peak that was slightly smaller than the peak that resulted from high-intensity premeal exercise, but much bigger than the postprandial glucose peak itself [32].

### 3.5. Comparison Studies

A training study that compared the effect of high-intensity exercise in fasted versus fed state (60 min after meal) in women found comparable improvements in body composition and muscle oxidative capacity. This study did not detect any significant change in insulin sensitivity in either group [40].

Three endurance training studies compared health effects, before meal versus after meal (two at 90 min after meal and one at 3 h after meal) all in healthy men [41–43]. Fat oxidation [41] and muscle adaptation did not change but GLUT4 [43] and glycogen content went up in the fasted training.

Glucose response to exercise at different intensities was compared in two studies on healthy men and men with type 2 diabetes [44, 45]. The lower intensity groups had lower glucose levels during the exercise [44] and throughout the subsequent 24 h period [45].

One study compared duration, 30 min every day versus 60 min every other day, at 90 min after meal in people with type 2 diabetes [46]. Both experiments showed comparable decreases in glycemia.

## 4. Discussion

Many of the 39 studies presented in Table 1 have straightforward graphical depictions of what happens to the typical glucose profiles of people with diabetes when exercise intervenes. These time versus glucose plots, viewed holistically, reveal the profound impact that timely exercise can have on blood glucose. First of all, the impact that exercise has on the glucose profile is highly dependent on the time elapsed between the start of the previous meal and the start of the exercise. Exercising at 30–45 min after meal at light to moderate intensity can reduce the postmeal glucose surge whereas exercising at a different time can increase the surge. Secondly, the effect appears to be direct and without any significant delay, as befits the source-sink relationship between exogenous glucose and physical activity. Thirdly, enough studies have accumulated to permit promising conjectures about what could be going on at the gross-physiological level, aside from the molecular processes involved. Clearly, energy balance has to be conserved: glucose molecules from the gut enter the blood stream and enrich the fuel supply and the excess thus generated is available to be used up in underwriting the physical activity rather than contributing to the surge. Which way the glycemic balance tilts depends on the rate of arrival of meal-derived glucose in the blood and the rate at which exercise draws away the fuel. Combine this process with the proclivity of hepatic glucose to enter the picture and fill any supply gaps, and what emerges is a simple, internally consistent view of the exercise-blood glucose dynamics.

### 4.1. Liver Glucose

During the prebreakfast hours counterregulation is up and active and elective physical activity uses liver glucose (along with fat and muscle glycogen) [8–21, 31–34]. Hepatic glucose involvement in fueling physical activity during early [22–25] and late [13, 32] postprandial periods depends upon the intensity of the exercise and also on how much glucose is present in the blood. Rising blood glucose during exercise indicates glucose arrival in the bloodstream in amounts greater than what the body expends. Premeal exercise resulted in postprandial glucose elevation [11–15] or, at high intensities, postexertion glucose elevation [31–34]. For light exercise during the early postprandial period [22–24] the overall blunting of the peak was suboptimal. Exercise during late postprandial period resulted in glycemic response similar to what was seen in the premeal studies [13, 32, 56]. Further, in four of the midpostprandial studies a transient rise of blood glucose was seen with increased intensity or duration of the activity signifying the arrival of endogenous glucose in the blood [17–19, 44]. In short, hepatic glucose output can be minimized by tactically adjusting the timing, intensity, and duration of the elective physical activity.

### 4.2. Meal-Derived Glucose

Exercising during the midpostprandial period led to decreased blood glucose levels [8–14, 17–19, 26–30, 44–46, 55–59]. Light to moderate aerobic exercise at 30 min after meal gave optimal blunting of the postmeal glucose peak [12, 26–28]. When exercise started at 30 min after meal, insulin-to-glucagon ratio went up and hepatic glucose output and fat oxidation remained suppressed during the activity [26, 28]. Glucose transport to muscles is increased via insulin-mediated or contraction-mediated transport systems or both. The body utilizes the excess glucose molecules appearing in the blood before they can accumulate and form the peak.

### 4.3. Exercise Intensity

The body at rest relies on free fatty acids for its major fuel needs. With rising intensity blood glucose and muscle glycogen enter the mix. Glucose (muscle glycogen and blood glucose) becomes the main fuel at high intensities (>80%  VO_2max_). Fat oxidation peaks around the intensity range of 50%–72%  VO_2max_ [60, 61]. At high-intensity exercise fat oxidation drops precipitously, catecholamine levels rise as much as 15-fold, and hepatic glucose output rises up to 8-fold [54, 62]. The extent of hepatic glucose production is determined by insulin-to-glucagon ratio during light to moderate exercise. Catecholamines play a comparable role when the activity is very intense. Improved insulin sensitivity observed with high-intensity exercise is brought about by the glycogen repletion that follows the glycogen depletion [15, 31, 35–38, 47–51, 54, 62, 63]. High-intensity exercise has mixed effects for diabetes patients: improved body composition, physical fitness, GLUT4 protein expression, mitochondrial function, oxygen capacity, and, to some extent, insulin sensitivity [15, 31, 47–52, 63]. Although the magnitude is modest the improvement in insulin sensitivity following high-intensity exercise is consistently observed [15, 31, 47–51, 53, 63]. Once the exercise training is stopped, the insulin sensitivity benefit disappears in about 60 hours [64]. In short, the insulin sensitivity improvement following high-intensity exercise is of slow onset, short-lived, and not nearly enough to adequately tame the glucose surges to come.

Markedly increased hepatic glucose output, on the other hand, resulted in a postexertion glucose elevation usually seen with intense premeal exercise [31–34]. Improved insulin sensitivity following muscle glycogen depletion can also lead to nocturnal hypoglycemia in people with type 1 diabetes [6, 47, 51]. The net effect of high-intensity premeal exercise on glycemia can be major glucose swings.

### 4.4. High-Intensity Interval Training

High-intensity interval training efficiently improves body composition, physical fitness [40, 47–52], and insulin sensitivity [47–51]. But it is not clear whether high-intensity interval training is appropriate for diabetes patients. Apart from the basic question of physical feasibility on the part of diabetes patients, exercise with intensity >80%  VO_2max_ comes with some negative effects: negligible fat oxidation, elevated catecholamines, and increased hepatic glucose output [54, 62, 63]. None of these is diabetes friendly, although fat oxidation is observed to go up following intense activity [53]. The high levels of catecholamines are not salutary to a population that can ill afford high blood pressure too. The worst effect of all is wide glucose swings: from postexertion glucose elevation [31–34] to nocturnal hypoglycemia [6, 47, 51].

High-intensity interval training has received much attention lately as an efficient way to deal with glucose surges [15, 47, 49]. According to the review by Kessler and colleagues high-intensity interval training for 8–12 months showed improvement in some cardiometabolic risk factors, but the reported effects on fasting glucose, HbA_1C_, and HDL cholesterol were inconsistent [48]. Lipids in general did not improve. Moreover, two studies found no improvement in HbA_1C_ in patients with type 2 and type 1 diabetes with high-intensity interval training [65, 66].

### 4.5. Energy Expenditure

Of the 5 studies at 30 min after meal that showed efficient blunting of the glucose peak, one was a brisk walk for 20 minutes [12]; two were at 55%  VO_2max_ for 45 min [26, 27]. The other two studies had durations of 60 min each and intensities of 50%  VO_2max_ and 71%  VO_2max_, respectively [19, 28]. It looked like the effectiveness of the exercise depended upon intensity and duration which together determine energy expenditure. Two studies, one at 45 min after meal [44] and the other at 60 min after meal [45], compared glucose responses to exercise at different intensities. In both cases the result showed that the lower intensity produced better glucose control. At relatively high intensities, glucose from endogenous sources rushing to meet the increased demand can lead to elevated glucose levels. For example, when the intensity was 50%  VO_2max_, glucose level did not rise during the 60 min duration of the exercise [28]. On the other hand, when the intensity rose to 71%  VO_2max_ glucose level went up 20 min into the activity [19].

The best tactic for taming glucose surges is to go with a light aerobic activity (<60%  VO_2max_) at 30 min after meal for up to 60 min [28]. At higher intensities duration may be reduced to 20–30 min [19]. Patients can fine-tune the intensity and duration guided by glucose monitoring. Those who do not have a glucometer can be trained with relative ease to keep the intensity at 60–65%  HR_max_ (HR_max_ is 220 − age) and do a brisk walk accordingly for about 30 min.

### 4.6. Resistance Plus Aerobic Exercise

Three studies demonstrated the role of postmeal resistance exercise in lowering glucose surges [7, 39, 67]. When Heden and colleagues used moderate intensity resistance exercise for 45 min starting at 45 min after meal, glucose levels came down during the exercise partially blunting the surge in obese patients with type 2 diabetes [39]. Another study showed that an acute bout of moderate resistance exercise 2.5 hours after meal prevalence of hyperglycemia was reduced by 35% over the next 24 hours in people with impaired glucose tolerance and in those with type 2 diabetes with or without insulin [67]. Yet another study used combined resistance and endurance exercise of moderate intensity during the postmeal surge to get hyperglycemia reduced by 39% during the 24 hours following the exercise bout [7]. Although combined training is better for glucose control (HbA_1C_) than resistance training or endurance training alone the data on these when the training was conducted at postmeal period are not available [68–73]. Resistance training 3 times a week [74] at midpostprandial period may have added benefits in controlling glucose surges.

### 4.7. Hypoglycemia

Transient, inconsequential hypoglycemia could strike when exercising in the late postprandial period [1, 7] and delayed nocturnal hypoglycemia [6, 47, 51] is a distinct possibility after intense premeal exercise in people with type 1 diabetes. Nelson and colleagues observed no hypoglycemia during the first 35 minutes of light exercise in both healthy people and people with type 1 diabetes when the activity started at 30 min after meal [26]. Praet and colleagues noted mild hypoglycemia, glucose concentration below 3.9 mmol/L, in 6 out of 11 subjects when the exercise was done during the late postprandial period [7]. Most diabetes patients can blunt the glucose peak and simultaneously minimize the risk of hypoglycemia by starting a bout of light to moderate physical activity at 30 minutes after meal. In the midpostprandial period the glucose still coming in from the gut will guard against hypoglycemia. Patients with diabetes can further fine-tune the intensity and duration with the help of glucometer readings.

## 5. Strengths and Limitations

Out of the 39 studies included in Table 1, 17 had sample sizes numbering in the single digits. Another limitation is the nonuniform categorizations used for exercise intensities in different studies included in this review.

In spite of the small sample sizes, a wide spectrum of metabolic conditions could be seen among the 615 subjects in the 39 studies. The actual numbers were 230 (type 2), 50 (type 1), 70 (obesity, prediabetes, metabolic syndrome, or insulin resistance), and 265 (healthy.) Twenty-one studies used control groups, whereas the others used a crossover design. All 39 studies used supervised exercise and all but two reported using standardized diets. The outcome measures in general could be seen across multiple studies, yielding consistent and reproducible results. Also on the positive side, the results of this review are the exposition of a few systematic problems bedeviling diabetes research. These are identified immediately below.

## 6. Looking Ahead

A valuable next step would be a lifestyle study to measure the effect on metabolic parameters of a timely postmeal exercise of light to moderate intensity starting 30 min after major meals. Also, it is useful to see the effect of adding a 10 min resistance exercise to the above lifestyle before the endurance exercise [68–72], 2-3 times a week [73].

Exercise timing is not specified in many studies and this makes it quite difficult to make sense of the results. Timing, intensity, frequency, and duration of the physical activity are needed to assess the full glucose response. Also, many long-term studies involving high-intensity interval training discuss improvements in insulin sensitivity without reporting the values of HbA_1C_ [47–51].

AUC calculations are not properly standardized. Incremental AUC, total AUC, and positive AUC as well as other arbitrarily defined AUC calculations have been reported, and it is difficult to compare the results. The abundance of misguided creativity seen here appears to be born of the notion that calculations can be used in such a manner that “less pronounced differences” between observed outcomes “would be easier to detect” [75]. Calculations are not microscopes, and calculation-dependent effects are not scientifically trustworthy. Sticking with the total AUC is the only legitimate option here.

## 7. Summary and Conclusions

Light to moderate exercise during the midpostprandial period preferentially uses meal-derived glucose and lowers blood glucose levels because insulin and glucose levels are high and, therefore, hepatic glucose output and fat oxidation are inhibited. To achieve the critical goal of blunting the postmeal glucose peak, it is best to start the aerobic exercise [<80%  VO_2max_] at 30 min after meal and continue for up to 60 min. Hypoglycemia risk is minimal with this approach. Exercising at any other time, before meal, early after meal, and late after meal, can trigger endogenous glucose production, resulting in elevated blood glucose levels. Although high-intensity (>80%  VO_2max_) exercise is effective in improving body composition, physical fitness, and insulin sensitivity its feasibility and efficacy in improving cardiometabolic markers for diabetes patients need to be established. This is because high-intensity exercise can offer wide fluctuations in glucose excursion: postexercise glucose elevation and delayed nocturnal hypoglycemia. There are indications that appending a bout of resistance training at moderate intensity (60%–80%  VO_2max_) to the daily endurance exercise 2-3 times a week is appropriate, if the patient is fit enough to undertake it.

We have come a long way with diabetes management, but the progress has not touched the majority of patients who live in developing countries and in the pockets of poverty in the West. Medications, medical care, glucometers, and choice diets are simply not accessible to the vast majority of diabetes sufferers in these parts of the world. They must fight the disease with physical activity and little else. Promoting a light to moderate, timely postmeal exercise after major meals would be of tremendous benefit universally to all with insulin resistance. Such a lifestyle practice would not violate any current guidelines: guidelines encourage exercise any time.

## Figures and Tables

**Table 1:** Glucose response to different exercise conditions: moderate exercise, before meal [8–21] and after meal [22–30]; high-intensity exercise, before meal [31–34] and after meal [35–39]; comparisons of training, fast versus fed [40–43], timings [44, 45], and durations [46].

Study	Subjects	Exercise protocol	Results
	T1D (type 1 diabetes); T2D (type 2 diabetes)	HIT (high-intensity interval training)	FFA (free fatty acid), PPG (postprandial glucose), Ra (rate of appearance), and AUC (area under the curve)
Gaudet-Savard et al. [8]	43 men with T2D	Light, before meal for 1 h versus after meal tested at 6 time intervals 0-1, 1-2, 2-3, 3-4, 4-5, and 5–8, 60% VO_2peak_	Exercise in fasted state is safe, no hypoglycemia; decrease in blood glucose depends on preexercise glucose level

Poirier et al. [9]	10 men with T2D	Light exercise, before meal versus 2 h after meal, both at 60% VO_2peak_ for 1 h	Moderate exercise in fasted state has minimal impact on blood glucose; exercise 2 h after meal decreases plasma glucose

Poirier et al. [10]	19 men with T2D	Light exercise, before meal for 1 h versus after meal tested at 6 time intervals 0-1, 1-2, 2-3, 3-4, 4-5, and 5–8, 60% VO_2peak_	Exercise in fasted state does not decrease blood glucose; blood glucose decreases with postprandial exercise, no clinical hypoglycemia is observed, and in postprandial state low blood sugar is seen

Derave et al. [11]	7 men with metabolic syndrome	Light exercise before meal, versus 1 h after meal, 60% VO_2peak_ for 45 min versus no exercise	Blunted glucose response with postmeal exercise, excessive glucose response with premeal exercise, and later meals unaffected

Colberg et al. [12]	12 men and women with T2D	Brisk walk, before meal versus 30 min after meal, for 20 min versus no exercise	Postdinner walking better for blunting postprandial glucose excursion and the postdinner glucose peak bigger with predinner exercise

DiPietro et al. [13]	10 prediabetes men and women	Light walks, 1 h after meal for 15 min each ×3 and 2.5 h and 4.5 h after meal (before dinner) for 45 min	Postmeal walks improve 24 h glycemia, there is no 24 h glucose improvement with predinner walk, and 3 bouts of 15 min postmeal walk are more effective than 45 min of morning or afternoon walk

Yamanouchi et al. [14]	6 patients with T1D	Premeal walk versus postmeal walk at 60 min after meal, 50% VO_2max_ for 30 min versus no walk	Glucose levels and glucose-AUC significantly lower only in the postmeal walking (premeal walk 17.8, postmeal walk 3.8, and no walk 11.8 h mM)

Francois et al. [15]	9 individuals with insulin resistance	Premeal HIT (three 10 min bouts) versus moderate premeal exercise, 60% VO_2max_ for 30 min	Premeal HIT exercise results in improved insulin sensitivity; moderate exercise leads to postprandial glucose elevation

Melton et al. [16]	16 prediabetes women	Moderate premeal exercise, 65% HR_max_ for 45 min versus no exercise	No effect on glucose, triglyceride, or oxidative stress

Kirwan et al. [17]	6 healthy women	Exercise before meal versus after meal (45 min), 60% VO_2peak_ to exhaustion	As insulin and glucose go up, FFA and glycerol are suppressed for 120 min of postmeal exercise, glucose is steady with premeal exercise for 120 minutes, and duration is not altered

Borer et al. [18]	9 healthy postmenopausal women	Premeal exercise, versus postmeal exercise (1 h) at 43% max effort, 2 bouts of 2 h each	Only prolonged light premeal exercise improves fasting glucose; FFA and D-3 hydroxybutyrate go up more during premeal exercise indicating liver glycogen depletion

Marmy-Conus et al. [19]	6 healthy men	Moderate premeal exercise versus moderate postmeal exercise starting 30 min after meal, 71% VO_2max_ for 60 min	Muscle glucose uptake increased, liver glucose output decreased by 62% with the postmeal exercise, and glucose level goes up 20 min into the exercise

Short et al. [20]	Study 2: 11 young adults	Moderate aerobic exercise 17 h before meal versus 1 h before meal, 75% VO_2peak_ for 45 min versus no exercise	Glucose-AUC 6% lower with the 1 h premeal trial within 3 h after the exercise, the effect not seen at 17 h after exercise

Oberlin et al. [21]	9 sedentary patients with T2D	Moderate premeal exercise 60–75% HR_max_ for 1 h versus no exercise	Glucose-AUC improved 15% after the 2nd meal

Høstmark et al. [22]	9 young and 10 middle-aged sedentary women, 10 young and 10 middle-aged trained women	Light bicycling starting 15 min after meal for 30 min versus no exercise	Light postmeal physical activity reduces blood glucose by a magnitude similar to that obtained by using drugs

Aadland and Høstmark [23]	9 healthy people	Very light intensity (VLI) and light intensity (LI) walk starting 15 min after meal for 30 min versus no walking	Both VLI and LI exercise blunted and delayed the rise in blood glucose

Nygaard et al. [24]	14 healthy women	Slow walking starting 15 min after meal for 15 min versus 40 min versus no walking	Even slow postmeal walking reduces postprandial glucose response to meal; this response is dose dependent on duration

Rasmussen et al. [25]	7 people with T1D	Bicycling starting 15 min after meal, 65% VO_2max_ for 30 min versus no exercise	Moderate postmeal exercise starting 15 min after meal for 30 min reduces blood glucose response by one-third

Nelson et al. [26]	9 people with T1D, 7 heathy people	Exercise starting 30 min after meal, 55% VO_2max_ for 45 min	Glycemic response to breakfast entirely normalized, symptomatic hypoglycemia seen after 35 min into exercise

Caron et al. [27]	8 people with T1D	Exercise starting 30 min after meal, 50% VO_2max_ for 45 min	Glucoregulation improves with strategically timed postmeal exercise

Shin et al. [28]	8 young healthy men	Exercise starting 30 min after meal, 50% VO_2max_ for 60 min	Higher insulin action decreases glucose, free fatty acid levels, and fat oxidation and increases growth hormone levels during exercise

Larsen et al. [29]	9 sedentary men with T2D	Exercise starting 45 min after meal, 53% VO_2max_ for 45 min versus no exercise versus diet	Moderate postmeal exercise decreases glycemia, the effect does not persist to affect subsequent meal, and the effect is similar to what follows decreased calorie intake

Van Dijk et al. [30]	60 T2D men (23 insulin-treated)	Endurance exercise, 2 h after meal, 35%–50% W_max_, for 45–60 min versus no exercise	With exercise glycemic control per continuous glucose monitoring improves throughout the subsequent day. HbA_1C_ is related to the magnitude of response to exercise

Kjaer et al. [31]	7 men with T2D and 7 healthy men	Single bout premeal HIT exercise at 100–110% VO_2max_	60 min of postexercise hyperglycemia in T2D followed by increased insulin effect on glucose disposal that is present 24 h after exercise. This has less therapeutic value in T2D

Kreisman et al. [32]	10 healthy men for premeal exercise and 8 healthy men for postmeal exercise	High-intensity exercise before meal versus 3 h after meal	Ra response to high-intensity exercise is preserved in postprandial exercise. Postexercise hyperglycemia is relatively reduced in postprandial exercise

Mitchell et al. [33]	8 T1D and 8 healthy	High-intensity premeal exercise at 80% VO_2max_	Postexercise hyperglycemia for 2 h, diabetes control deteriorates with intense premeal exercise

Yale et al. [34]	8 lean and 12 obese people	High-intensity premeal exercise to exhaustion	Obese people had greater postexercise insulin resistance

Larsen et al. [35]	8 sedentary men with T2D	High-intensity postmeal exercise, 45 min after meal, 98% VO_2max_ versus no exercise	Intense postmeal exercise does not deteriorate glucose homeostasis, effect related to energy expenditure, and the effect does not help lunch

Gillen et al. [36]	7 adults with T2D	HIT 90 min after meal versus no exercise	HIT exercise at 90 min after meal reduces postprandial hyperglycemia up to 24 h

Little et al. [37]	10 inactive obese men	HIT 2 h after breakfast versus continuous moderate intensity exercise, 65% VO_2max_ for 30 min versus no exercise	No effect on lunch. PPG-AUC for dinner and for next breakfast better for HIT, absolute AUC and absolute spikes not different

Szewieczek et al. [38]	14 T2D and 14 healthy	HIT 2 h after meal versus no exercise	Hyperglycemia reduced during recovery period with HIT

Heden et al. [39]	13 obese T2D patients	Resistance exercise (RE) 45 min after dinner, before dinner versus no exercise	Predinner RE improves postprandial glucose concentration; postdinner exercise improves both glucose and TAG concentrations

Gillen et al. [40]	16 women	HIT, fasted versus fed, starting at 60 min, 3/week for 6 weeks	HIT is time efficient, fed versus fasted: both improve body composition and muscle oxidative capacity

De Bock et al. [41]	20 healthy men	Endurance training, 10 fasted versus 10 fed starting at 90 min ×3/week for 6 weeks at 75% VO_2peak_	Fat oxidation similar, glycogen breakdown less in fasted training

van Proeyen et al. [42]	27 healthy men	Endurance training, 10 fasted versus 10 fed starting at 90 min, ×4/week for 6 weeks versus 7 no training	Fasted training is (slightly) more potent in muscle adaptations

Nybo et al. [43]	15 healthy men	Endurance training, 7 fasted versus 8 fed starting at 3 h postprandial ×4/week for 8 weeks at 70–85% VO_2peak_	Muscular adaptations similar in fast versus fed training except GLUT4 and glycogen content more in fasted training

Achten and Jeukendrup [44]	8 healthy men	45 min after meal 40%, 65%, 80% VO_2max_ for 20 min	Insulin peaks at 30 min after meal; insulin and glucose levels decrease in 10 min similarly (then glucose level goes up for 80%)

Manders et al. [45]	9 sedentary men with T2D	Starting 60 min after meal light (35% W_max_) for 60 min versus moderate intensity (70% W_max_) exercise for 30 min	Light exercise as opposed to moderate exercise reduces hyperglycemia throughout the subsequent 24 h, prevalence of hyperglycemia 50% versus 19%

Van Dijk et al. [46]	30 patients with T2D	90 min after meal, 50% W_max_ 30 min every day versus 60 min every other day versus no exercise	Hyperglycemia lower in both exercise regimens
